# An Advanced Sensor for Particles in Gases Using Dynamic Light Scattering in Air as Solvent

**DOI:** 10.3390/s21155115

**Published:** 2021-07-28

**Authors:** Dan Chicea, Cristian Leca, Sorin Olaru, Liana Maria Chicea

**Affiliations:** 1 Research Center for Complex Physical Systems, Lucian Blaga University of Sibiu, Dr. Ion Ratiu Str., No. 5-7, 550012 Sibiu, Romania; cristian.leca@continental-corporation.com (C.L.); sorin.olaru@continental-corporation.com (S.O.); liana.chicea@ulbsibiu.ro (L.M.C.); 2 Continental Automotive Systems Srl, Salzburg Str. 8, 550018 Sibiu, Romania

**Keywords:** dynamic light scattering, particle sizing, advanced fire sensor, air DLS

## Abstract

Dynamic Light Scattering is a technique currently used to assess the particle size and size distribution by processing the scattered light intensity. Typically, the particles to be investigated are suspended in a liquid solvent. An analysis of the particular conditions required to perform a light scattering experiment on particles in air is presented in detail, together with a simple experimental setup and the data processing procedure. The results reveal that such an experiment is possible and using the setup and the procedure, both simplified to extreme, enables the design of an advanced sensor for particles and fumes that can output the average size of the particles in air.

## 1. Introduction

The detection, measurement and analysis of particles suspended in air is of great importance in fields like environment monitoring, health, pollution, combustion engines, the automotive field, fire detection, meteorology, and many others.

Particles in suspension in air are also called Aerosols or Particulate Matter or Airborne particles.

The size of particles suspended in air is up to 100 µm. Larger particles are not suspended, and their falling velocity is usually higher than 1 m/s.

There are several methods and sensors for analyzing the particles suspended in air. A few important methods are explained briefly in the next paragraph.

Mechanical methods consist of mechanically collecting particles in suspension from air. This can be done either with filters or with a centrifuge. If filters are used, a fan forces the air to pass through a filter which collects particles from air. The particles attached to the filter are examined with a microscope and very detailed information about the particles can be obtained. This method is simple and straightforward, but at the same time, it is laborious and time consuming. A faster, simplified mechanical method is to use selective filters with descending sizes of holes. A version of this method is the gravimetric method described in [1]: a clean filter is weighed, then a large amount of air with particles is filtered through it. After filtering the high volume of air, the filter is weighed again. The mass difference divided by the volume of filtered air is the mass concentration of particles.

Another method for mechanical collection of suspended particles is with a centrifuge. The force applied on a particle inside a centrifuge is thousands of times higher than its weight in normal gravity. For example, a 20 µm particle falls with 1 cm/s in air in normal gravity 1 g. The same particle will have a velocity of 32 cm/s in a centrifuge with 1000× *g* or 1 m/s in a large centrifuge with 10,000× *g*. Smaller particles that do not settle down in normal gravity can be separated in a centrifuge. Then, particles attached on the circumference of the centrifuge can be analyzed with a microscope.

Mechanical methods are precise and accurate, but they are limited to laboratory measurements. They are not suitable for automated analyses or high-volume measurements or continuous monitoring of air.

A different method is by ionizing the particles suspended in air. In the middle of the twentieth century, the first smoke sensor with a radioisotope and ionization chamber appeared [2]. Such sensors include a very small amount (0.3 µg) of Americium 241, which ionizes molecules of air inside the ionization chamber. Americium 241 is preferred because its radiation is 1% gamma and 99% alpha, which has high ionization power and also can be easily shielded. A voltage is applied between two electrodes in the chamber and the current between electrodes is monitored. If particles of smoke are inside the chamber, ions will adhere to them, and the current between electrodes will change. Therefore, the smoke can be detected. Such sensors are not very reliable and are being used less and less frequently. Sometimes they are used in conjunction with other type of sensors, especially because sensors with ionization are more sensitive for small-sized particles, unlike optical sensors, which are more sensitive for large particles.

Ionization of air molecules can be performed by Corona discharge instead of radioactivity. Article [3] explains in detail an experimental device for analyzing the particles in the exhaust gas from a diesel engine.

The signal generated by a smoke sensor with ionization depends on both the size and density of particles; therefore, neither of these parameters can be measured. The size could be measured if the density of particles was known, following a calibration with particles of known size. However, these conditions cannot be achieved for a commercial sensor.

Optical sensors are the most widely used for detecting and analyzing particles in air. A light source illuminates a volume of air. The particles inside the illuminated volume scatter the light. A light sensor measures either the transmitted light or the scattered light, and the generated electrical signal is amplified and analyzed to obtain information about the particles.

Sensors that count particles have a light source (LED or laser) and lens that focuses the light beam to the size of a particle. A thin air flow with particles flows between light source and a photodetector, in the region where the light is focused. Each particle that passes between the light source and the photodetector will produce a pulse in the signal from the photodetector. The pulses are counted, and the density of particles can be calculated. Some detectors have additional electronics and software for analyzing each pulse and estimating the size of each particle, but the precision is very low, and they can only distinguish between dust and smoke. One of the most advanced such sensors is produced by Sensirion and is described in [1].

Optical smoke sensors are installed in most public buildings and some houses. An LED and a photodiode are placed inside an optical chamber. They are arranged so that neither direct nor reflected light from the LED can arrive on the photodetector. Particles suspended in air that enter in the chamber scatter the light, and a certain amount of scattered light is detected by the photodiode.

Sensors with scattered light are low cost, small and reliable. They are used for detection of smoke or dust, but they cannot measure the parameters of particles. The level of signal generated by the photodetector is highly dependent on particle density, size, shape, and color; therefore, none of these parameters can be measured separately.

In recent years, more sophisticated sensors with scattered light have been developed. For example, Siemens developed a smoke sensor with two light sources placed so that the photodetector measures light scattered in two directions [4]. Scattered light from large particles is more intense for small angles and weak for large angles. Scattered light from small particles is isotropic. The described sensor can measure the scattered light in two directions and some information about the size of particles is available. This sensor cannot measure the size of particles, it can only distinguish between smoldering fire (large particles) and open fire (small particles) and is more reliable for detection of all types of fire.

Nephelometers are used for precise measurement of concentrations of particles suspended in a fluid. They are, basically, optical sensors with scattered light at a 90° angle. Although they are expensive and sensitive, they cannot directly determine the concentration of particles. As mentioned before, the scattered light depends on the concentration, size, shape, and color of particles. The size, shape and color must be known in advance in order to measure the concentration. A nephelometer must be calibrated for a known particulate before measuring the concentration in atmosphere.

The most advanced nephelometers can measure the size distribution of particles. This objective can be achieved by Static Light Scattering (SLS) method. The nephelometers manufactured by Air Photon [5] or TSI [6] measure the scattered light for three wavelengths and for a scattering angle from 7° to 170°. The achieved data from three photomultipliers (for three wavelengths) is processed to obtain the size distribution and density of particles. These instruments are expensive, heavy (8 kg), and include many mobile parts and sensitive optical components.

Optical sensors with extinction measure the intensity of direct light from an LED or laser. When particles are between the source of light and detector, the intensity of light on photodetector is lower. Most sensors have an optical chamber and the distance between the light source and the photodetector is a few centimeters. Alternatively, the distance is several meters. In this case, a laser beam aims a photodetector that is located several meters away. The monitored area is thus much larger, but false detections may occur more frequently.

A few other methods are used occasionally for analyzing particulate matter. These methods have been applied in laboratory only.

A frame of four CCD linear sensors can scan all particles that pass through the frame. Detailed information about particles can be achieved after data processing [7].

Another type of particle analyzer uses the noise produced by falling particles on a metal sheet. The sound is captured by a microphone and analyzed, and details regarding particles can be obtained [8].

The Dynamic Light Scattering (DLS) method has been used since 1964 for measuring the size of particles suspended in liquid. The DLS method needs the same basic components as SLS or Scattered Light Sensors or nephelometers: a photodetector and a light source that must be monochromatic and coherent, i.e., a laser. The essential difference is the measured parameter of light. DLS requires the frequency of the scattered light, while all the other methods require the intensity of scattered light. The monochromatic light scattered from the particles create an interference image on the photodetector. The intensity of light on photodetector is the result of random phases of light from illuminated particles. However, the particles are moving continuously due to Brownian motion, and therefore, the phase and intensity of light on photodetector is variable. The velocity of particles in Brownian motion depends on the size of particles. The frequency of the signal generated by the photodetector is processed by a computer and the size of particles can be calculated. Table 1 Presents an overview of the most important methods for measuring particles suspended in air.

To our knowledge, the DLS method has never been applied to particles in gas. This article proves the possibility of measuring the size of particles in suspension in air using a small, wearable, calibration free, low-cost device based on DLS in air. Details are presented further on.

## 2. Materials and Methods

### 2.1. A Brief Overview on the DLS Data Processing Procedure

The DLS procedure makes use of a coherent light beam focused on particles suspended in a solvent, usually a liquid solvent [9,10,11,12,13]. The light scattered by particles is coherent as well; therefore, an interference image is produced [14] and the interference intensity of light can be measured by a detector and recorded using a data acquisition system (DAS hereafter). The resulting recorded data are called a Time Series in DLS and are processed in a quite simple manner to obtain the average diameter of the suspended particles [9,10,15]. There are several approaches, each based on certain approximations and assumptions, that can lead to the calculation of the particle size distribution, such as CONTIN [16,17] or Maximum Entropy algorithms [18,19].

CONTIN is based on the inverse Laplace transform [16,17], and introduces regularization to reduce the number of mathematical structural items in expressions. The inverse Laplace transform applied to numerical data requires filtering [20], and it is computation intensive [21]. Moreover, the inverse Laplace transform may lead to ambiguous results, this being an ill-posed mathematical problem. CONTIN counteracts this problem by introducing regularization, which is driven by a parameter with large influence on the resulting solution. Selecting the proper value of this parameter is problematic, cannot be done entirely automatically, and the choosing an inadequate value of the parameter can lead to completely incorrect solutions.

The maximum entropy method [18,19] is an improvement of the fitting method. It consists of assigning entropy to the solutions and then searching for the solution with maximum entropy. The fitting in the maximum entropy method is also computationally intensive.

Both methods address the purpose of the work reported in this article, which is to find a simple procedure that can be applied to process data for an advanced sensor for particles in air. Therefore, we will present here a simple DLS procedure that can be used to process DLS time series generated from particles in air, a procedure that can output the average size of the nano and microparticles, thus being an advanced sensor, while at the same time being a simple, low-cost device.

**Figure 1:**
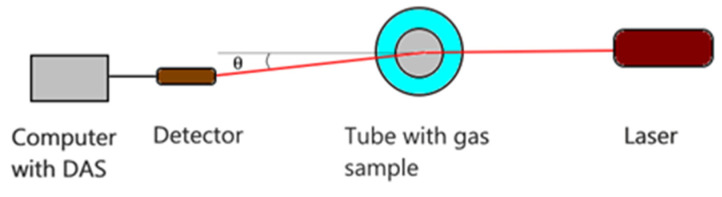
The experimental setup.

A schematic of the basic DLS setup is depicted in Figure 1. The light source, which must be coherent, can be either a He-Ne laser or a laser diode. The typical wavelength for these light sources is 633 nm. The scattering angle θ is variable, as will be explained in the next subsection. The side displacement can be adjusted to have the proper scattering angle. The samples consist of micro and nanoparticles suspended in air. The distance D between sample and detector can be adjusted so that the average speckle size will match the size of the detector (as good as possible).

The DLS time series consists of values recorded by DAS with a certain sampling rate f. This means the intensity of light is recorded at time intervals Δt = 1/f. As stated in [22,23], the width of the autocorrelation of the intensity time series is proportional to the diffusion coefficient, which depends on diameter of scattering centers (SC hereafter).

The early reports [9,10] and the more recent improvements [11,12,24] prove that the frequency spectrum is related to the Probability Density Function (hereafter PDF). The frequency spectrum (FS hereafter) is related to the autocorrelation of a process, as demonstrated by the Khinchin–Kolmogorov theorem [13,25]. An alternative version is described later.

The FS can be described analytically using the Lorentzian line S(f) (1).

(1) $$S\left(f\right)=a_{0}\frac{a_{1}}{{\left(2\pi f\right)}^{2}+a_{1}{}^{2}}$$

Two parameters, a_0_ and a_1_, are included in the Lorentzian line S(f). The values of the parameters in Equation (1) must be determined so that the Lorentzian line S(f) best describes the FS computed with the recorded time series. The parameters can be determined using a minimization procedure [15,26]. Then, the radius can be calculated using Equations (2) and (3):

(2) $$R=\frac{2k_{B}{\mathrm{Tq}}^{2}}{6{\pi \eta a}_{1}}$$

where q is the modulus of the scattering vector:

(3) $$q=\frac{4\pi n}{\lambda }\sin\frac{\theta }{2}$$

In Equations (2) and (3), R is the average radius of the particles in suspension, k_B_ is Boltzmann’s constant, T is the absolute temperature of the solvent, η is the dynamic viscosity of the solvent, n is the refractive index of the solvent, λ is the wavelength of the laser beam, and θ is the recording angle [15,26].

### 2.2. Challenges of Performing DLS Measurements for Particles in Gases

A detailed analysis of the particular conditions required to perform DLS on particles suspended in air was carried out, and the results were presented in our previous work, reported in [27]; therefore, only a brief summary is presented here.

Equation (1) describes the shape of the FS of the DLS time series of particles in a solvent of a particular temperature scattering angle. To plot the expected shape of the ideal FS, a_1_ can be calculated by reverting Equation (2).

**Figure 2:**
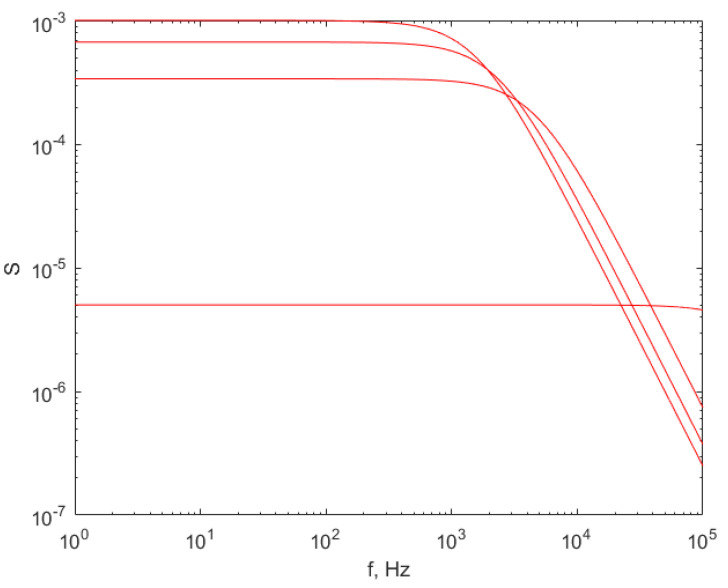
The simulated FS for particles diameters in the set: 5, 338, 672 and 1000 nm at a scattering angle of 90°, with air at 100 °C as solvent. The lower curve is the FS for the lowest diameter, 5 nm, while the upper curve is for the largest diameter, 1000 nm. The sampling rate was 2 × 10^5^ samples/s.

**Figure 3:**
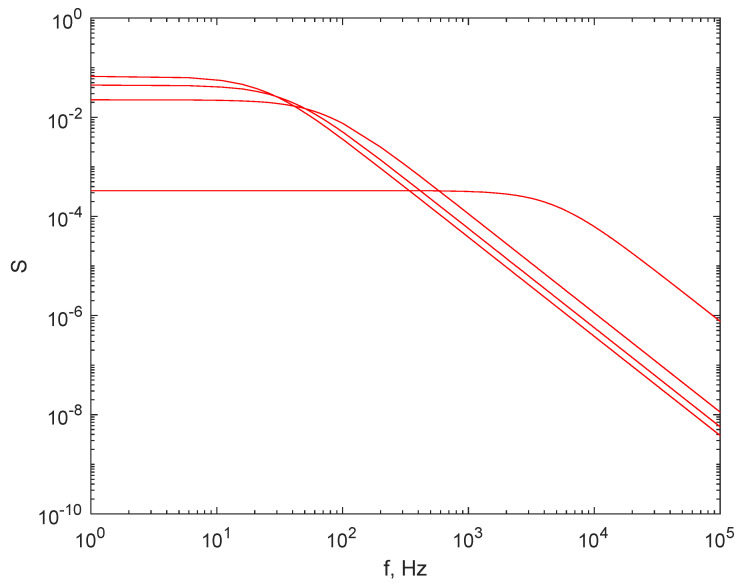
The simulated FS for the set of diameters at a scattering angle of 10°. The lower curve is the FS for the lowest diameter, 5 nm, while the upper curve is for the largest diameter, 1000 nm. The sampling rate was of 2 × 10^5^ samples/s.

If SCs are suspended in water, the parameters are: temperature = 20 °C, n = 1.33 and η = 1.02 × 10^−3^ daP. If the particles are suspended in air at 100 °C, the parameters are roughly two orders of magnitude smaller: n = 1 and η = 2.1704 × 10^−5^ daP. This affects the parameter a_1_ for the same radius of the particles. Equation (2) reveals that for the same radius R, a_1_ is inversely proportional to η; therefore, a decrease in η will increase the a_1_ parameter accordingly, so that the turnover point (frequency) in the plot of the FS versus frequency, as in Figure 2 and Figure 3, will be shifted toward a higher frequency [27]. For a successful processing of a DLS time series, higher data acquisition sampling rates are necessary, necessitating more expensive equipment for light detection and data acquisition, thus exceeding the scope of the intended device, which is a relatively low-cost sensor using common electronics. By common electronics we mean electronic devices and components manufactured in large series rather than custom, small series, or application-specific integrated circuits (ASIC). Such devices are used in audio devices and personal computers, including audio preamplifiers and computer sound cards, whether internal cards or external USB sound cards.

Reference [27] presents in detail the results of the simulation of the frequency spectrum of a time series acquired using a sampling rate of 200 k samples/s for particles with the diameter in the range 10–1510 nm, for a set of angles between 10° and 90°. We will present here a simulation of the expected FS for particles with the diameters of: 5, 338, 672 and 1000 nm.

The typical scattering angle for most DLS experiments is 90°, mainly because in terms of FS, the plateau of the logarithmic–logarithmic plot is stretched over a wider frequency range, and therefore, assessing the turnover point, the a_1_ parameter thus is more precise. The frequency spectrum, simulated for the diameters mentioned above, is illustrated in Figure 2.

Figure 2 reveals that the turnover point (frequency) in the lowest curve, corresponding to SC diameter 5 nm, is beyond the highest limit of the frequency range in the plot; therefore, the least squares fitting method will not identify a_1_ in Equation (1). Consequently, the correct radius of the smaller-sized nanoparticles cannot be determined using the parameters mentioned above [27].

Reference [27] illustrates in detail plots of the expected FS produced by particles in the above-mentioned set over an extended range of scattering angles θ, and it can be easily noted from the succession of plots that the plateau becomes narrower and stretches over a smaller frequency range as the recording angle decreases. The plots for smaller scattering angles in [27], like 5°, suggest that DLS is possible in the air for relatively low sampling rates of 10^5^ samples/s. Although this sampling rate may appear low, such sampling rates are not easy to achieve with relatively low-cost electronics, as defined above.

If we consider the class of low-cost electronics, we have in mind sampling rates of up to of 4.4 × 10^4^ samples/s. Moreover, we can imagine using a sound card for a PC or laptop that is a low-cost and good-quality DAS. A sampling rate of 4.4 × 10^4^ samples/s is usual for acquisition of high-fidelity sound with frequency up to 22 kHz. This turned out to be the case, as sound cards for computers have been constantly improved over decades and have maintained a low price as they are produced in very large number. Moreover, a sound card is included in almost every desktop or laptop. Common sound cards have an analog–digital convertor with a resolution of up to 32 bits. However, sound cards must be used with a certain caution, provided that there is no spectral attenuation from the detector to the input, because sometimes filters are applied in sound cards or computers for sound recording.

Figure 3, below, depicts the simulated frequency spectrum for the same diameter set previously mentioned, assuming the same sampling rate of 2 × 10^5^ samples/s, recorded at a scattering angle of 10°.

If the scattering angle is decreased to 10°, we notice that a successful fit of the Lorentzian line to the computed FS might be possible; therefore, a_1_ and the diameter of the particles can be determined, because the turnover point of the line is now inside the frequency range of the spectrum computed using the Fast Fourier Transform (FFT) algorithm, which will be 0–2.2 × 10^4^ Hz should a sampling rate of 4.4 × 10^4^ Hz be used. Moreover, the turnover points of the lines corresponding to different diameters of particles are clearly separated from each other in Figure 3; thus, the least squares method can be applied for fitting with a reasonable precision.

### 2.3. Details on DLS Experimental Setup and Data Processing Procedure

The experimental setup that was used to record time series for DLS for particles in air is illustrated in Figure 1. The coherent light source was an He-Ne laser with the usual wavelength of 633 nm. The scattering angle θ was chosen to be 10°, as was decided during the FS simulations. The detector consisted of a silicon PIN photodiode SFH203P and a transimpedance amplifier. The acquisition and recording were carried out using the sound card of a PC at a sampling rate of 44,100 samples/s. Amplitudes were extracted later on and the DLS time series was processed by the data processing procedure, as it is described further on.

Air viscosity strongly depends on temperature; therefore, the temperature measurement is mandatory for each DLS time series recording. The temperature was measured using a digital thermometer with the temperature sensor in the transparent tube right above the laser beam. The coefficient of dynamic viscosity η was calculated using the Sutherland correlation [28], which expresses its dependence on the absolute temperature of an ideal gas, based on kinetic theory of ideal gases.

The time series was recorded for 30 s each time. The FS was calculated using the Fast Fourier Transform (FFT) algorithm [29] and only 2^20^ = 1,048,576 data were used, simply because the FFT algorithm uses data of the type 2^n^. If more data are supplied, the function pads the rest of the values with 0 values to match the number 2^(n+1)^, with the direct consequence that a much higher amplitude is indicated for very small frequencies than the real one.

**Figure 4:**
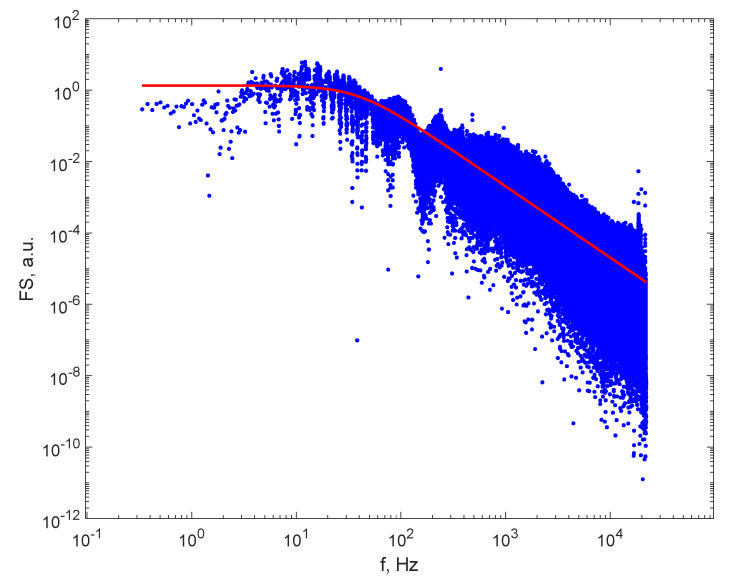
The FS after filtering (blue dots) and the Lorentzian line (the continuous line) for the particles in smoke from paper burning with flame.

An FS of one of the recorded DLS time series is illustrated in Figure 4. It can be seen that the rollover point is inside the frequency range, and this fact enables the assessment of a_1_ and subsequently the diameter of the particles. The FS of the recorded time series contains noise that is visible on plots as spikes around some frequencies, with amplitudes much higher than the amplitudes in the rest of the spectrum. Therefore, filtration is required. Such plots are presented in [26,30], therefore not repeated here. A filtering procedure was applied after the FS had been computed, as described in [29,30], which removed a bandwidth of 2.5 Hz centered on 50 Hz and the upper harmonics, as 50 Hz is the power grid frequency and noise at this frequency is present in all recordings.

Moreover, a normalization of the FS was applied, as well, as described in [30], and this is crucial for DLS on particles suspended in gas. The intensity of the scattered light strongly depends on both the number density of the particles and on the particle size, through the scattering parameter g [14,15]; therefore, different samples will produce FSs with considerably different amplitudes. A least squares minimization procedure was used to find the parameters a_0_ and a_1_, that is, the parameters of the Lorentzian line were found that best described the FS computed on the DLS time series. The fitting procedure stops, among other criteria, when either of the parameters changes by less than a predetermined quantity. Fitting is more accurate when both parameters have comparable values, or at least the same order of magnitude. The parameter a_1_ is in the range of tens to thousands; therefore, the normalization of the FS was achieved by multiplying all amplitudes in a set in the FS to cause the height of the plateau to be on the order of magnitude of several thousands.

After filtering the FS spectrum and normalizing it, the Lorentzian line was fitted to the spectrum as described in Section 2.1; a_0_ and a_1_ were determined followed by the calculation of radii of the particles using Equation (2) and the diameters.

### 2.4. Error Calculation

First of all, we should verify that the fluid flowing around the object, SC in this work, is in the Stokes regime. The autocorrelation of a DLS time series, or the FS depend on the diffusion coefficient. If the fluid flowing around the object is in the Stokes regime, the drag is described by the Stokes equation, and Equation (2) is correct. The Knudsen number describes the fluid flowing regime. A discussion on the Knudsen number for DLS on particles in gas is presented at the end of Section 3, together with the approximation of particles with a narrow size distribution. Examination of the plot of the residual of the least squares fitting provides insight into this matter; therefore, this possible systematic error will be discussed at the end of Section 3, which presents the FS and the fit of the Lorentzian line.

Moving further, on the assumption that Equation (2) is perfectly accurate, we can derive the relative error when assessing the particle radius R by replacing Equation (3) in Equation (2) and by writing the logarithm of R as a first step, in Equation (4).

(4) $$R=\frac{16{\pi k}_{B}n^{2}}{{\eta a}_{1}\lambda^{2}}\cdot {\mathrm{Tsin}}^{2}\frac{\theta }{2}$$

If we consider all the constants to be grouped as one factor, the differential of that factor will be null. If we consider that the quantities that we measured and which, therefore, were sources of errors, were the thermodynamic temperature T and the measuring angle θ, the logarithm of R is:

(5) $$\ln\left(R\right)=\ln\frac{16{\pi k}_{B}n^{2}}{{\eta a}_{1}\lambda^{2}}+\mathrm{lnT}+2\ln\left(\sin\frac{\theta }{2}\right)$$

If we differentiate Equation (5) and we consider dT and dθ to be the experimental errors in measuring those quantities, under the assumption of the most unfavorable situation when errors sum, we obtain Equation (6):

(6) $$\varepsilon_{R}=\frac{\Delta R}{R}=0+\frac{\Delta T}{T}+\frac{1}{\tan\left(\frac{\theta }{2}\right)}\cdot \Delta \theta$$

Considering an error of 3 K for temperature, the detector–tube distance and the diameter of the transparent tube, we found that the relative error was quite large, as large as 31%. While the error is quite large, it is still in line with the purpose of the work described in this paper, which is to describe a simple setup and data processing procedure for a sensor, not a procedure to increase the precision with respect to the existing DLS procedures mentioned in the introductory section. A column describing the error when assessing particle diameter using our experimental setup and data processing procedure is presented in Table 2, together with the average diameters for the samples used to test the procedure.

## 3. Results and Discussion

Several materials were ignited with different flame regimes to produce smoke and particles and were used as targets of the laser beam in the experimental setup, as depicted in Figure 1. The samples that produced particles are presented in Table 2.

Figure 4 illustrates the FS computed for the DLS time series recorded on smoke from paper burning with flame as a source of particles, after filtering the grid noise and harmonics and after fitting the Lorentzian line to the FS. We notice that the 50 Hz noise frequency and the harmonics were removed. The red, continuous line represents the Lorentzian line (Equation (1)) plotted with the parameter of best fit, where a_1_ was equal to 244.2 Hz, corresponding to a larger average particle diameter in the fumes, of 565 nm.

Figure 4 also reveals that the line fits reasonably well to the computed FS, confirming that the approximation of having monodisperse particles in the sample is acceptable.

**Figure 5:**
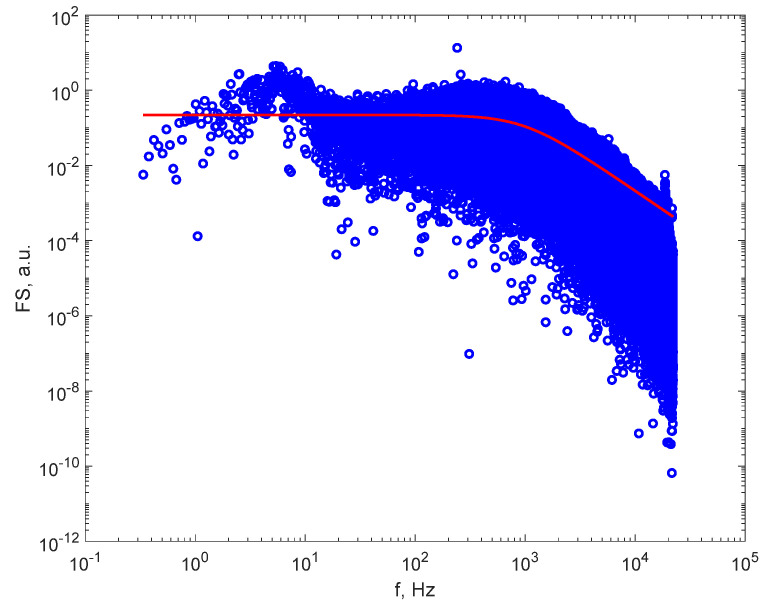
The FS after filtering (blue dots) and the Lorentzian line (the continuous line) for the particles of cigarette smoke.

Figure 5 illustrates the FS computed for the DLS time series recording on cigarette smoke, following the same data processing procedure, after filtering the grid noise and harmonics and after fitting the Lorentzian line to the FS. The red, continuous line representing the Lorentzian line was plotted with the parameter of best fit, which, for this sample, was a_1_ equal to 6166.0 Hz, corresponding to an average diameter of the particles in the cigarette smoke of 22 nm. We notice that the monodisperse particle size distribution approximation holds on this sample, which has the smallest average particle diameters, as well, because the line fits the experimental FS reasonably well.

Moreover, we notice that the turnover point in the plot with logarithmic distancing of the ticks on the axes of the plots lies within the frequency range, as expected from the simulation presented in Section 2.2, for a smaller recording angle of 10°, as used when recording the DLS time series, proving that DLS in air, at a low acquisition rate, can be successfully accomplished.

The same aspect of the FS and the line fit to it can be noticed on the FS for the DLS time series recorded on the particles produced by a smoldering wax candle wick, where the average diameter was 78 nm, and for particles produced by smoldering paper, where the computed average diameter was 15 nm. The plots of the computed FS with the plotted Lorentzian line of best fit are not presented here, though, as they do not provide any additional insight.

**Figure 6:**
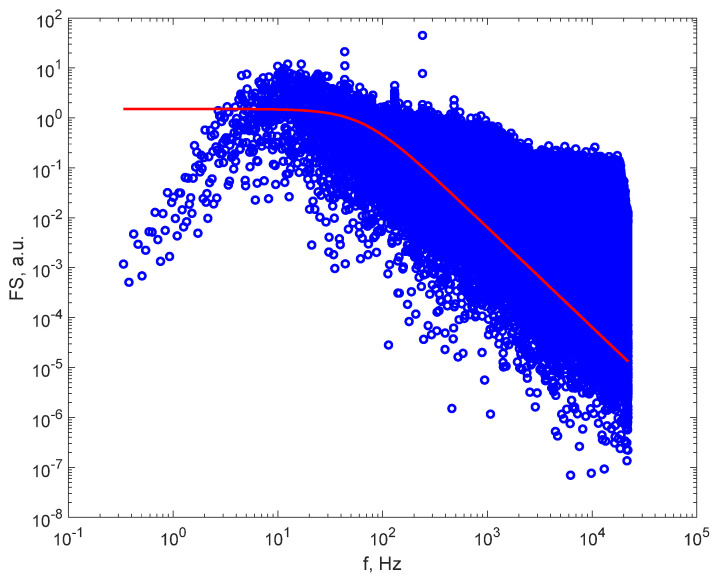
The FS after filtering (blue dots) and the Lorentzian line (the continuous line) for the particles in air produced by a nebulizer.

So far, the samples that were analyzed and presented suggest that DLS time series recorded for particles in air as solvent with relatively low data acquisition rates can be processed using the approximation whereby the particles are assumed to possess a monodisperse or narrow size distribution. The procedure must be tested on samples with wider known particle size distributions, and the results must be compared with the real size distribution. Such a source of particles in air is a nebulizer that uses a small compressor to disperse medical drugs in aerosol particles. Figure 6 illustrates the FS computed for the DLS time series recording on aerosol water droplets used as a sample, following the same data processing procedure. The red, continuous line represents the Lorentzian line, and was plotted with the parameters of the best fit, which, for this sample, was a_1_ equal to 410.0 Hz, corresponding to an average diameter of the particles in aerosol droplets of 336 nm. The aerosol droplets had a wider distribution this time, with a maximum diameter of 2.6 μm. We notice that the low frequency part of the FS does not appear to be a plateau, and that the whole FS appears to be a sum of FSs recorded for monosized particles. The fit indicated an average diameter of 336 nm.

Even so, the fit indicated an average diameter consistent with the size distribution of the droplets, despite their wider distribution. Moreover, the size of the particles assessed using the procedure described in Section 3 are consistent with the generic size of different particles, as reported in [31], which states that oil smoke has a particle size in the range 0.03–1 µm, tobacco smoke is in the range 0.01–4 µm, and burning wood with flame is in the range 0.2–3 µm, which we assume to be comparable with burning paper. The tobacco smoke we used was not produced by inhaling it, but by smoldering; therefore, the size of the particles is smaller than that reported in [31].

**Figure 7:**
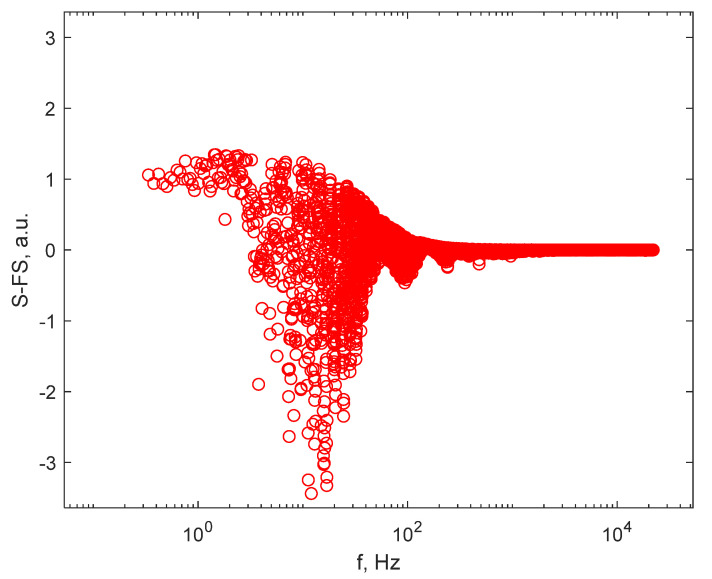
The residual for the particles in smoke from paper burning with flame.

To better judge the hypothesis that the particle distribution can be approximated as being unimodal, and, therefore, the FS ca be approximated by the Lorentzian line described by Equation (1), the residual of the least squares fit with the Lorentzian line should be plotted. A plot of the residual on the data recorded on particles in smoke from paper burning with flame, presented in Figure 4, is illustrated in Figure 7. A double logarithmic plot, as in Figure 4 through Figure 6, would be optimal, but the residual has both positive and negative values, and therefore the frequency axis can only be represented by values displayed on a logarithmic scale. Examining the residual of the least squares fit, it can be noticed that the values are both positive and negative, but they are not quite randomly distributed around 0; rather, certain patterns are present. This is an indication that the particle distribution is not perfectly unimodal, but rather presents a certain width. Even so, the device and the procedure produce the average diameter in the DLS sense, and the average diameter is a reasonable measure of the size of the particles suspended in gas.

Another concern related to the procedure presented in this paper is related to Equations (2) and (3), which are derived from a fluid flowing around the particles in Brownian motion in the Stokes regime. To verify the flow regime, the mean free paths of the gas molecules λ should be compared with the characteristic dimension of the object, that is, the particles that scatter light in the case of this paper. The Knudsen number Kn is a dimensionless number defined as the ratio of the molecular mean free path length to a characteristic dimension of the object that is moving through the fluid, the average diameter of the particles.

(7) $$\lambda =\frac{1}{\sqrt{2}{\pi d}_{\mathrm{gas}}{}^{2}N/V}$$

In Equation (7), N/V is the number concentration of molecules per unit volume and d_gas_ is the diameter of the gas molecules. If we consider a Boltzman gas and we replace N/V we find for Kn:

(8) $$\mathrm{Kn}=\frac{k_{B}T}{\sqrt{2}{\pi d}_{\mathrm{gas}}{}^{2}\mathrm{pd}}$$

where p is the gas pressure, T is the thermodynamic temperature and d is the diameter of the particle in suspension. The pressure was 101,100 Pa, the temperature was 22 °C, except for the particles from the paper burning with flame, where the temperature was 83 °C in the beam area of the tube. The Knudsen number was calculated for the particles we measured and is displayed as column 5 of Table 2. Reference [32] states that for Kn < 0.1 there is a slip flow, and for Kn between 0. 1 and 10 the flow regime is transitional to free molecular flow. Examining Table 2, it can be noticed that the particles that were investigated using this DLS in the gas procedure undergo a Brownian motion, where the flowing of the fluid is in a transitional regime, not in a slip flow or continuum flow regime; therefore, Equation (2) should be considered an approximation. The procedure described above to be used for assessing the average particle diameters, together with the simple experimental setup should be viewed as an advanced sensor, not as a very precise procedure intended to replace existing DLS particle sizers. Nevertheless, the procedure outputs an average diameter, with a systematic error, yet reproducible and without requiring calibration, which places the device and the procedure in the advanced sensor category. Even so, the average diameters found using the procedure were in the range of the diameters reported in the literature, as pointed out before.

A very precise measurement would hardly be possible, as explained in the Introduction section. To make a precise measurement, a large quantity of particles is required, and harvesting this from smoke would take time, during which the burning regime would need to be kept at the same parameters. An alternative would be Atomic Force Microscopy (AFM), as described in papers like [33,34,35], but it is affected by systematic errors as well, due to the finite radius of the AFM cantilever tip, as clearly explained in [36] and many other papers.

The DLS technique has as an output the hydrodynamic diameter, not the physical diameter. For this reason, the diameter described above should be considered the hydrodynamic diameter (3). Particles of nonspherical shape, like nanorods or irregular shapes, diffuse in air. If they scatter a coherent light beam, the wavelets interfere, producing a DLS time series. If the time series is analyzed with the procedure describes in Section 2.2, an average hydrodynamic diameter in the DLS sense [37] is produced. Such a diameter should be understood as the diameter of the spherical particles that diffuse in air as the real particles do.

## 4. Conclusions

The work reported here describes a procedure that is based on the DLS technique with data processing carried out in a simple manner, by fitting the theoretically expected shape of the FS, which is the Lorentzian line described by Equation (1), to the FS computed on the experimentally recorded DLS time series. This simple data processing procedure is not altered by any assumption that might be used to assess the diameter distribution, as pointed out in Section 2.1, because it has as an output the average diameter, as previously described. The work described in this manuscript stands as a proof of concept for the possibility of using a very simple device, made of a tube and a laser source, even a laser diode, for recording a small-angle scattered-light time series using a common computer sound card and processing it. The whole sensor, including the simple experimental setup and the simplified data processing procedure, does not intend to replace or to be as precise as the commercially available DLS particle sizers, and the limitations of this sensor are clearly stated in this paper. Yet, such a simple yet advanced sensor could be programmed to indicate the presence of particles bigger than a triggering size. This setup can be used to indicate that a material is burning with flame rather than smoldering. It can be used as a sensor in very clean areas, like microfabrication laboratories or in sterile medical chambers, where it can indicate the presence of viruses or bacteria. The sensor could be used to assess the average diameter of the fog particles, as well, which might be crucial in assessing the LiDAR efficiency in automobile traffic safety devices. This advanced sensor cannot indicate whether the micron size particles that were detected were bacteria or viruses rather than airborne dust, though.

## Figures and Tables

**Table 1:** An overview of the most important methods for measuring particles suspended in air.

Basic Method	Variant	Measured Parameter of Particles			Measurement Time	Accuracy	Sensor Complexity	
		Number Volume concentration	Mass Volume concentration	Size of particles			Size	Cost
Mechanical	With filters	yes	yes	yes	Very long	good	Large	Medium
	With centrifuge	yes	yes	yes	Very long	good	Large	High
	Gravimetric	no	yes	no	Very long	Very good	Large	High
Ionization	With radioactive materials	yes	no	no	Short	Medium	Medium	Medium
	With high voltage	yes	no	no	Short	Medium	Medium	Medium
Optical	Particles counting	yes	no	no	Short	Very good	Small	Medium
	Transmitted light intensity	yes	no	no	Short	Poor	Small	Medium
	Scattered light intensity	yes	no	no	Short	Poor	Small	Low
	SLS	no	yes	yes	Short	Medium	Large	High
	DLS Scattered light frequency	no	no	yes	Short	Good	Small	Medium

**Table 2:** The samples that were analyzed, with the a_1_ parameter and the average diameter.

Sample	a_1_, Hz	Average Diameter d, nm	Δd,nm	Kn
Smoke from paper burning with flame	244.2	565	175	0.14
Wick of a wax candle, smoldering	1771.4	78	24	0.83
Nebulizer	410.0	336	104	0.19
Cigarette smoke	6166.0	22	7	3.0
Smoke from paper, smoldering	9393.1	15	5	4.39
